# The genome sequence of an ichneumon wasp, *Clistopyga incitator* (Fabricius, 1793)

**DOI:** 10.12688/wellcomeopenres.19302.1

**Published:** 2023-05-12

**Authors:** Lucy Broad, Gavin R. Broad, Natural History Museum Genome Acquisition Lab, Darwin Tree of Life Barcoding collective, Wellcome Sanger Institute Tree of Life programme, Wellcome Sanger Institute Scientific Operations: DNA Pipelines collective, Tree of Life Core Informatics collective, Darwin Tree of Life Consortium

**Affiliations:** 1 Independent researcher, Tonbridge, England, UK; 2 Natural History Museum, London, England, UK

**Keywords:** Clistopyga incitator, ichneumon wasp, genome sequence, chromosomal, Hymenoptera

## Abstract

We present a genome assembly from an individual female *Clistopyga incitator* (an ichneumon wasp; Arthropoda; Insecta; Hymenoptera; Ichneumonidae). The genome sequence is 260.7 megabases in span. Most of the assembly is scaffolded into 21 chromosomal pseudomolecules. The mitochondrial genome has also been assembled and is 25.0 kilobases in length.

## Species taxonomy

Eukaryota; Metazoa; Ecdysozoa; Arthropoda; Hexapoda; Insecta; Pterygota; Neoptera; Endopterygota; Hymenoptera; Apocrita; Parasitoida; Ichneumonoidea; Ichneumonidae; Pimplinae; Ephialtini; *Clistopyga*; *Clistopyga incitator* (Fabricius, 1793) (NCBI:txid494138).

## Background

While most ichneumonids, or Darwin wasps, are parasitoids of immature stages of insects, a few different groups have evolved to take advantage of spiders and their egg sacs. The most species-rich radiation of spider parasitoids and predators within Ichneumonidae is within the subfamily Pimplinae ( Gauld *et al.*, 2002; Gauld & Dubois, 2006; Matsumoto, 2016). *Clistopyga* is a Pimpline genus found across much of the world with several species known to be predators within spider egg sacs ( Nielsen, 1929) and some presumed or confirmed to be parasitoids of adult spiders ( Fritzén & Sääksjärvi, 2016) in their silk nests.

*Clistopyga* females are readily recognised by their upcurved ovipositors. They are often attractively patterned, with *C. incitator* usually being recognisable on colour pattern, with several creamy white marks on the head and thorax and a red and black mesosoma (thorax plus the propodeum). The upcurved ovipositor is used to penetrate dense silken egg sacs of spiders to lay eggs. *Clistopyga incitator* has been reared from the egg sac of *Segestria senoculata* (Linnaeus), a spider which spins dense silken tubes, often in stone walls. The sequenced specimen of *C. incitator* was found walking on a stone wall. The ovipositor of *C. incitator* might have another function too. In a closely related (unidentified) species, tiny ‘teeth’ on the lower ovipositor valves pull silk threads when the ovipositor is being withdrawn from the silk nest. Repeated movements act to close up the hole made by the ovipositing female, with the ovipositor acting in a manner analogous to a felting needle ( Fritzén & Sääksjärvi, 2016).

*Clistopyga incitator* is a reasonably common, widespread species, found across Europe and temperate Asia, for example, Kasparyan DR, 2007; Yu, van Achterberg and Horstmann, 2016. At least in southern Britain, there seem to be two broods per year ( Fitton *et al.*, 1988).

The subfamily Pimplinae has been the focus of several studies aiming to understand how an amazing variety of life history strategies has evolved in one lineage, for example Wahl and Gauld, 1998; Gauld, Wahl and Broad, 2002; Klopfstein *et al.*, 2019. However, the Pimplinae which belongs to an assemblage (the pimpliformes) which seems to have initially rapidly diversified in the Jurassic, might not be monophyletic ( Spasojevic *et al.*, 2021). As the first complete assembly for a species of the subfamily Pimplinae, the genome of *Clistopyga incitator* should pave the way for new approaches to investigating the evolution of different parasitoid life histories in relatively closely related taxa.

## Genome sequence report

**Figure 1.**
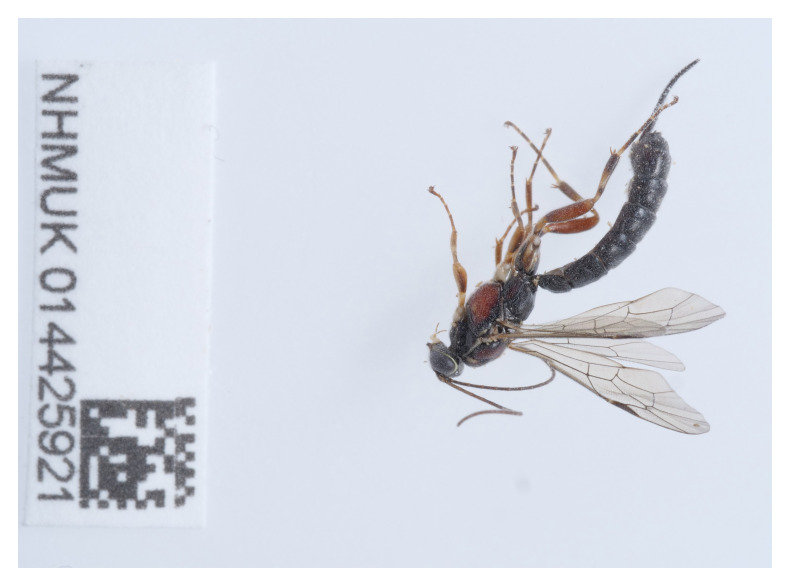
Photograph of the *Clistopyga incitator* (iyCliInci1) specimen used for genome sequencing.

The genome was sequenced from one female *Clistopyga incitator* ( Figure 1) collected from Bowcombe Creek, UK (latitude 50.28, longitude –3.76). A total of 68-fold coverage in Pacific Biosciences single-molecule HiFi long reads was generated. Primary assembly contigs were scaffolded with chromosome conformation Hi-C data. Manual assembly curation corrected 61 missing joins or mis-joins and removed 10 haplotypic duplications, reducing the assembly length by 2.32% and the scaffold number by 50%, and increasing the scaffold N50 by 12.45%.

**Figure 2.**
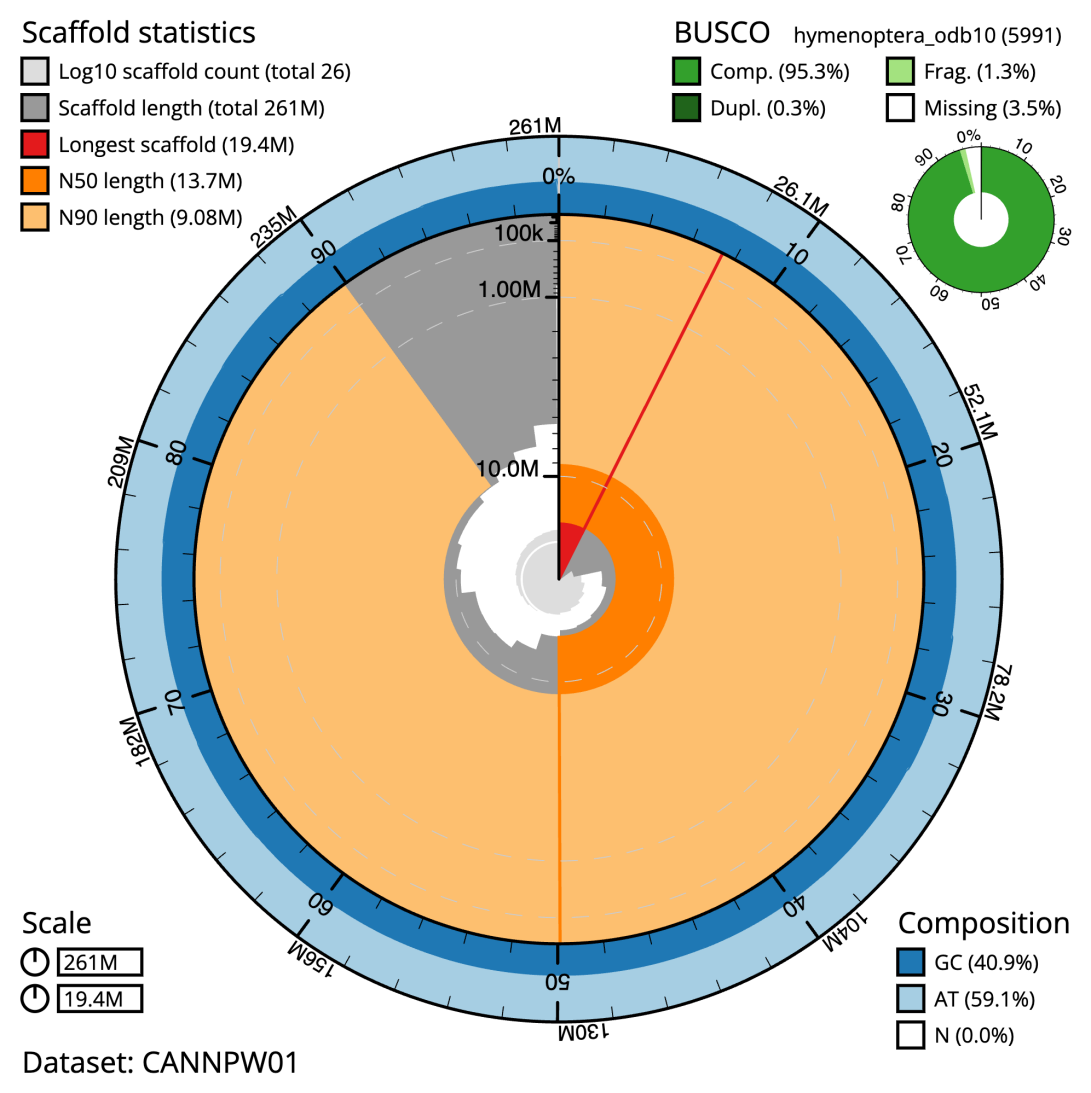
Genome assembly of *Clistopyga incitator*, iyCliInci1.1: metrics. The BlobToolKit Snailplot shows N50 metrics and BUSCO gene completeness. The main plot is divided into 1,000 size-ordered bins around the circumference with each bin representing 0.1% of the 260,700,937 bp assembly. The distribution of scaffold lengths is shown in dark grey with the plot radius scaled to the longest scaffold present in the assembly (19,385,043 bp, shown in red). Orange and pale-orange arcs show the N50 and N90 scaffold lengths (13,682,479 and 9,078,478 bp), respectively. The pale grey spiral shows the cumulative scaffold count on a log scale with white scale lines showing successive orders of magnitude. The blue and pale-blue area around the outside of the plot shows the distribution of GC, AT and N percentages in the same bins as the inner plot. A summary of complete, fragmented, duplicated and missing BUSCO genes in the hymenoptera_odb10 set is shown in the top right. An interactive version of this figure is available at https://blobtoolkit.genomehubs.org/view/iyCliInci1.1/dataset/CANNPW01/snail.

The final assembly has a total length of 260.7 Mb in 26 sequence scaffolds with a scaffold N50 of 13.7 Mb ( Table 1). Most (99.7%) of the assembly sequence was assigned to 21 chromosomal-level scaffolds. Chromosome-scale scaffolds confirmed by the Hi-C data are named in order of size ( Figure 2– Figure 5; Table 2). While not fully phased, the assembly deposited is of one haplotype. Contigs corresponding to the second haplotype have also been deposited. The mitochondrial genome was also assembled and can be found as a contig within the multifasta file of the genome submission.

**Table 1.** Genome data for *Clistopyga incitator*, iyCliInci1.1.

Project accession data		
Assembly identifier	iyCliInci1.1	
Species	*Clistopyga incitator*	
Specimen	iyCliInci1	
NCBI taxonomy ID	494138	
BioProject	PRJEB55729	
BioSample ID	SAMEA110044016	
Isolate information	iyCliInci1, female: head and thorax (genome sequencing and Hi-C scaffolding)	
Assembly metrics *		*Benchmark*
Consensus quality (QV)	65	*≥ 50*
*k*-mer completeness	100%	*≥ 95%*
BUSCO **	C:95.3%[S:95.0%,D:0.3%], F:1.3%,M:3.5%,n:5,991	*C ≥ 95%*
Percentage of assembly mapped to chromosomes	99.7%	*≥ 95%*
Sex chromosomes	Not applicable	*localised homologous pairs*
Organelles	Mitochondrial genome assembled	*complete single alleles*
Raw data accessions		
PacificBiosciences SEQUEL II	ERR10168721	
Hi-C Illumina	ERR10149549	
Genome assembly		
Assembly accession	GCA_947507545.1	
*Accession of alternate haplotype*	GCA_947507575.1	
Span (Mb)	260.7	
Number of contigs	183	
Contig N50 length (Mb)	2.4	
Number of scaffolds	26	
Scaffold N50 length (Mb)	13.7	
Longest scaffold (Mb)	19.4	

*Assembly metric benchmarks are adapted from column VGP-2020 of “Table 1: Proposed standards and metrics for defining genome assembly quality” from ( Rhie *et al.*, 2021). **BUSCO scores based on the hymenoptera_odb10 BUSCO set using v5.3.2. C = complete [S = single copy, D = duplicated], F = fragmented, M = missing, n = number of orthologues in comparison. A full set of BUSCO scores is available at https://blobtoolkit.genomehubs.org/view/iyCliInci1.1/dataset/CANNPW01/busco.

**Figure 3.**
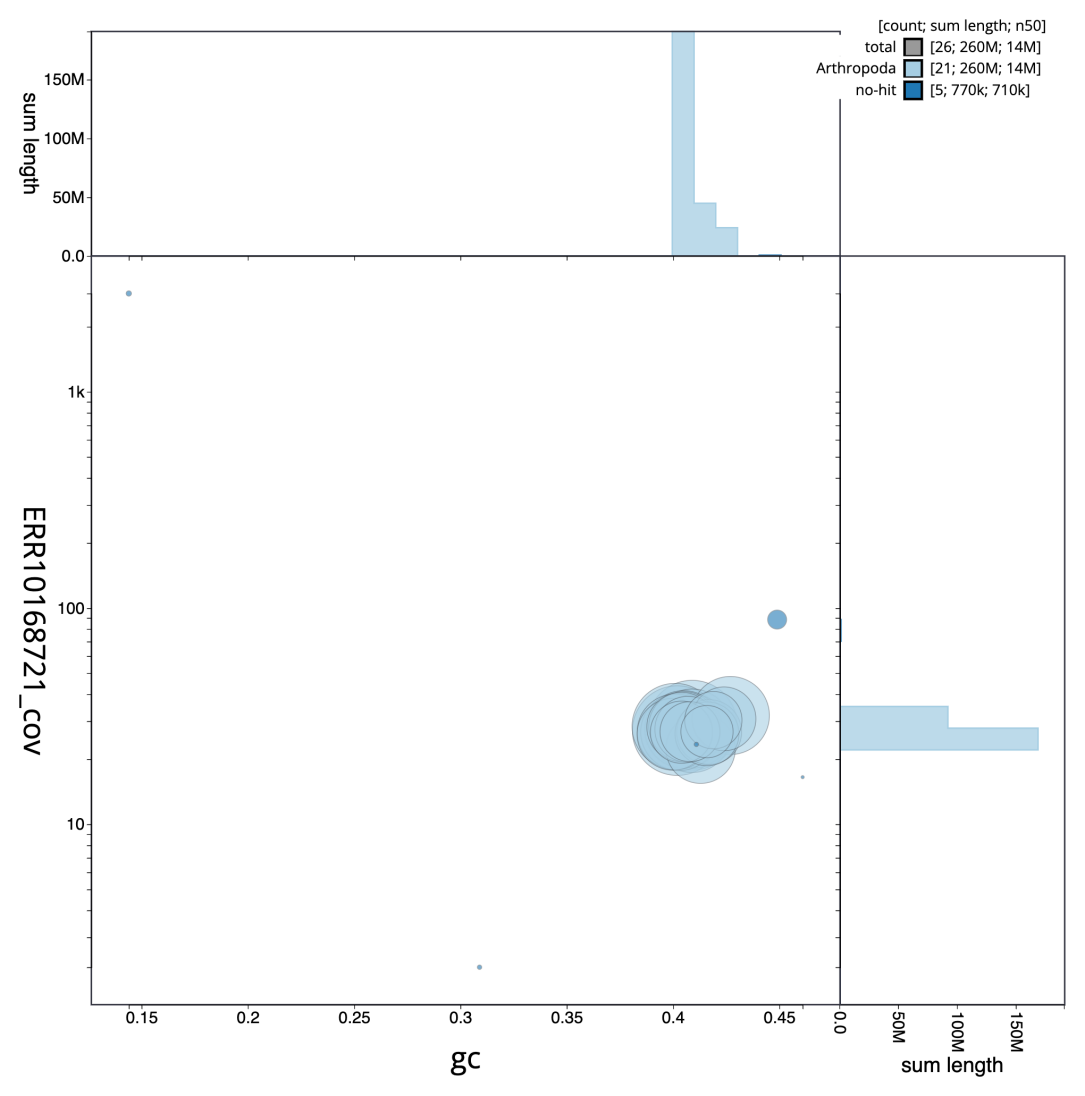
Genome assembly of *Clistopyga incitator*, iyCliInci1.1: BlobToolKit GC-coverage plot. Scaffolds are coloured by phylum. Circles are sized in proportion to scaffold length. Histograms show the distribution of scaffold length sum along each axis. An interactive version of this figure is available at https://blobtoolkit.genomehubs.org/view/iyCliInci1.1/dataset/CANNPW01/blob.

**Figure 4.**
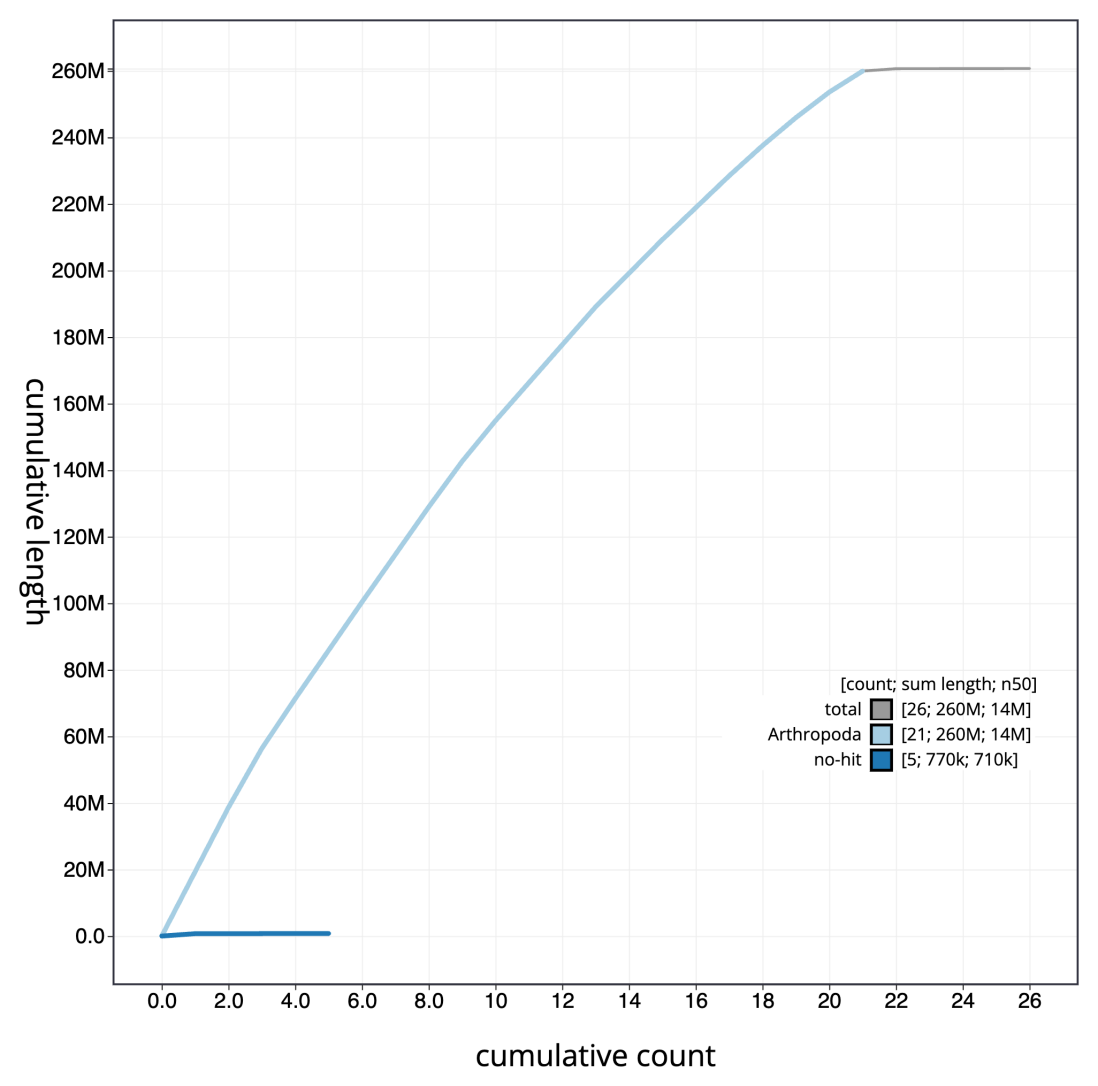
Genome assembly of *Clistopyga incitator*, iyCliInci1.1: BlobToolKit cumulative sequence plot. The grey line shows cumulative length for all scaffolds. Coloured lines show cumulative lengths of scaffolds assigned to each phylum using the buscogenes taxrule. An interactive version of this figure is available at https://blobtoolkit.genomehubs.org/view/iyCliInci1.1/dataset/CANNPW01/cumulative.

**Figure 5.**
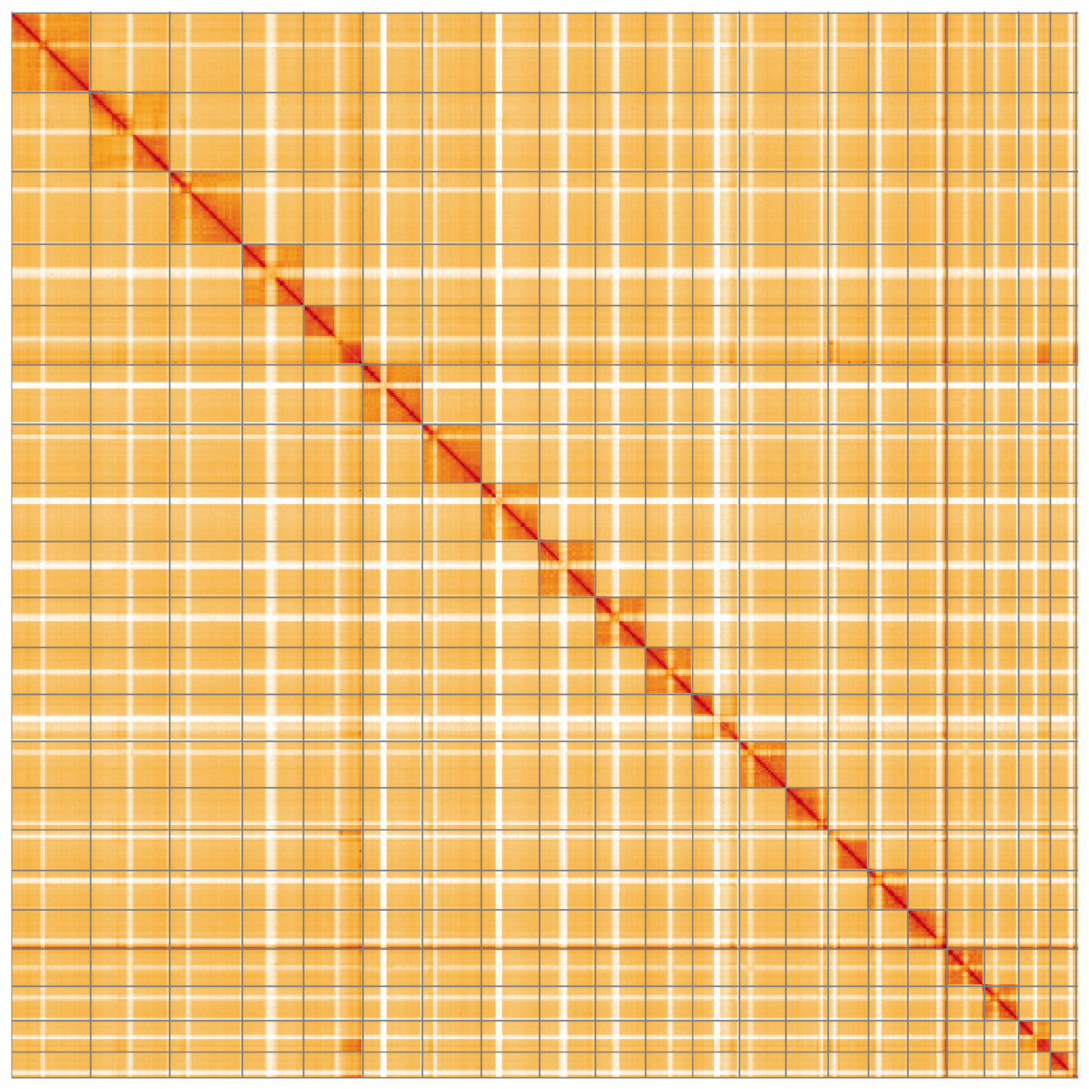
Genome assembly of *Clistopyga incitator*, iyCliInci1.1: Hi-C contact map of the iyCliInci1.1 assembly, visualised using HiGlass. Chromosomes are shown in order of size from left to right and top to bottom. An interactive version of this figure may be viewed at https://genome-note-higlass.tol.sanger.ac.uk/l/?d=Kh-LVKesSN6ytn8mcd0U6Q.

**Table 2.** Chromosomal pseudomolecules in the genome assembly of *Clistopyga incitator*, iyCliInci1.

INSDC accession	Chromosome	Size (Mb)	GC%
OX382171.1	1	19.39	40.9
OX382172.1	2	19.27	40.2
OX382173.1	3	17.73	40.1
OX382174.1	4	14.97	40.9
OX382175.1	5	14.51	42.7
OX382176.1	6	14.5	40.9
OX382177.1	7	14.34	40.5
OX382178.1	8	14.23	40.3
OX382179.1	9	13.68	40.1
OX382180.1	10	12.24	40
OX382181.1	11	11.46	40.5
OX382182.1	12	11.39	41.3
OX382183.1	13	11.37	40.4
OX382184.1	14	10.28	40.7
OX382185.1	15	9.92	41.7
OX382186.1	16	9.59	41.6
OX382187.1	17	9.57	42.4
OX382188.1	18	9.08	40.4
OX382189.1	19	8.41	40.8
OX382190.1	20	7.67	41.9
OX382191.1	21	6.34	41.6
OX382192.1	MT	0.02	14.4

The estimated Quality Value (QV) of the final assembly is 65 with *k*-mer completeness of 100%, and the assembly has a BUSCO v5.3.2 completeness of 95.3% (single = 95%, duplicated = 0.3%), using the hymenoptera_odb10 reference set ( *n* = 5,991).

Metadata for specimens, spectral estimates, sequencing runs, contaminants and pre-curation assembly statistics can be found at https://links.tol.sanger.ac.uk/species/494138 .

## Methods

### Sample acquisition and nucleic acid extraction

A female *Clistopyga incitator* specimen (iyCliInci1) was collected from Bowcombe Creek, UK (latitude 50.28, longitude –3.76) on 20 August 2021. The specimen was collected by hand by Lucy Broad (private address), and then identified by Gavin Broad (Natural History Museum) and frozen at –80°C.

The sample was prepared at the Tree of Life laboratory, Wellcome Sanger Institute (WSI). The iyCliInci1 sample was weighed and dissected on dry ice with tissue set aside for Hi-C sequencing. Head and thorax tissue of iyCliInci1 was disrupted using a Nippi Powermasher fitted with a BioMasher pestle. DNA was extracted at the WSI Scientific Operations core using the Qiagen MagAttract HMW DNA kit, according to the manufacturer’s instructions.

### Sequencing

Pacific Biosciences HiFi circular consensus DNA sequencing libraries were constructed according to the manufacturers’ instructions. DNA sequencing was performed by the Scientific Operations core at the WSI on the Pacific Biosciences SEQUEL II (HiFi) instrument. Hi-C data were also generated from tissue of iyCliInci1 using the Arima v2 kit and sequenced on the Illumina NovaSeq 6000 instrument.

### Genome assembly, curation and evaluation

Assembly was carried out with Hifiasm ( Cheng *et al.*, 2021) and haplotypic duplication was identified and removed with purge_dups ( Guan *et al.*, 2020). The assembly was then scaffolded with Hi-C data ( Rao *et al.*, 2014) using YaHS ( Zhou *et al.*, 2023). The assembly was checked for contamination as described previously ( Howe *et al.*, 2021). Manual curation was performed using HiGlass ( Kerpedjiev *et al.*, 2018) and Pretext ( Harry, 2022). The mitochondrial genome was assembled using MitoHiFi ( Uliano-Silva *et al.*, 2022), which runs MitoFinder ( Allio *et al.*, 2020) or MITOS ( Bernt *et al.*, 2013) and uses these annotations to select the final mitochondrial contig and to ensure the general quality of the sequence. To evaluate the assembly, MerquryFK was used to estimate consensus quality (QV) scores and *k*-mer completeness ( Rhie *et al.*, 2020). The genome was analysed within the BlobToolKit environment ( Challis *et al.*, 2020) and BUSCO scores ( Manni *et al.*, 2021; Simão *et al.*, 2015) were calculated. Table 3 contains a list of software tool versions and sources.

**Table 3.** Software tools: versions and sources.

Software tool	Version	Source
BlobToolKit	4.0.7	https://github.com/blobtoolkit/blobtoolkit
BUSCO	5.3.2	https://gitlab.com/ezlab/busco
Hifiasm	0.16.1-r375	https://github.com/chhylp123/hifiasm
HiGlass	1.11.6	https://github.com/higlass/higlass
Merqury	MerquryFK	https://github.com/thegenemyers/MERQURY.FK
MitoHiFi	2	https://github.com/marcelauliano/MitoHiFi
PretextView	0.2	https://github.com/wtsi-hpag/PretextView
purge_dups	1.2.3	https://github.com/dfguan/purge_dups
YaHS	yahs-1.1.91eebc2	https://github.com/c-zhou/yahs

### Ethics and compliance issues

The materials that have contributed to this genome note have been supplied by a Darwin Tree of Life Partner. The submission of materials by a Darwin Tree of Life Partner is subject to the Darwin Tree of Life Project Sampling Code of Practice. By agreeing with and signing up to the Sampling Code of Practice, the Darwin Tree of Life Partner agrees they will meet the legal and ethical requirements and standards set out within this document in respect of all samples acquired for, and supplied to, the Darwin Tree of Life Project. All efforts are undertaken to minimise the suffering of animals used for sequencing. Each transfer of samples is further undertaken according to a Research Collaboration Agreement or Material Transfer Agreement entered into by the Darwin Tree of Life Partner, Genome Research Limited (operating as the Wellcome Sanger Institute), and in some circumstances other Darwin Tree of Life collaborators.

## Data Availability

European Nucleotide Archive: *Clistopyga incitator*. Accession number PRJEB55729; https://identifiers.org/ena.embl/PRJEB55729 . ( Wellcome Sanger Institute, 2022) The genome sequence is released openly for reuse. The *Clistopyga incitator* genome sequencing initiative is part of the Darwin Tree of Life (DToL) project. All raw sequence data and the assembly have been deposited in INSDC databases. The genome will be annotated using available RNA-Seq data and presented through the Ensembl pipeline at the European Bioinformatics Institute. Raw data and assembly accession identifiers are reported in Table 1.

## Author information

Members of the Natural History Museum Genome Acquisition Lab are listed here: https://doi.org/10.5281/zenodo.4790042.

Members of the Darwin Tree of Life Barcoding collective are listed here: https://doi.org/10.5281/zenodo.4893703.

Members of the Wellcome Sanger Institute Tree of Life programme are listed here: https://doi.org/10.5281/zenodo.4783585.

Members of Wellcome Sanger Institute Scientific Operations: DNA Pipelines collective are listed here: https://doi.org/10.5281/zenodo.4790455.

Members of the Tree of Life Core Informatics collective are listed here: https://doi.org/10.5281/zenodo.5013541.

Members of the Darwin Tree of Life Consortium are listed here: https://doi.org/10.5281/zenodo.4783558.
