# Supplementary material for: The genome sequence of an ichneumon wasp, Clistopyga incitator (Fabricius, 1793)
Source: Wellcome Open Res. 2023 May 12;8:215. [Version 1] doi: 10.12688/wellcomeopenres.19302.1 (PMC11191966; doi:10.12688/wellcomeopenres.19302.1)
Supplement: Peer review history

# Reviewer report: Reviewer response for version 1

**DOI:** 10.21956/wellcomeopenres.21385.r85283

**Published:** 2024-06-18

**Authors:** Diego Galvão Pádua

**Affiliations:** 1 Centro de investigacion de Estudios Avanzados del Maule, Universidad Catolica del Maule, Talca, Maule, Chile

Broad and Broad propose the genome sequence of a Darwin wasp, Clistopyga incitator (Fabricius, 1793) (Pimplinae). This sequence, which will be in a free access database, will be used for future studies using molecular tools.

 I consider that the MS can be indexed with minor corrections and I present my observations and suggestions below:

 Title and abstract: I suggest replacing ichneumon wasp with Darwin wasp.

 Backgrounf: " *Clistopyga* females are readily recognised by their upcurved ovipositors." Except females of C. henryi species-group and C. isayae species-group.

 Figure 1: The specimen is upside down.

Are sufficient details of methods and materials provided to allow replication by others?

Yes

Is the rationale for creating the dataset(s) clearly described?

Yes

Are the datasets clearly presented in a useable and accessible format?

Yes

Are the protocols appropriate and is the work technically sound?

Yes

Reviewer Expertise:

Systematics of Darwin wasps.

I confirm that I have read this submission and believe that I have an appropriate level of expertise to confirm that it is of an acceptable scientific standard.

# Reviewer report: Reviewer response for version 1

**DOI:** 10.21956/wellcomeopenres.21385.r74767

**Published:** 2024-02-26

**Authors:** Shyaman Jayasundara

**Affiliations:** 1 Purdue University, West Lafayette, Indiana, USA

This note presents comprehensive details regarding the genome assembly of an ichneumon wasp, *Clistopyga incitator*. The authors have employed proper techniques and tools for the assembly. The note is well-written, and the methodology is straightforward and clear.

 Minor comment:

 The parameters and configurations used for the tools/software are not provided. Including them along with the scripts will increase reproducibility.

Are sufficient details of methods and materials provided to allow replication by others?

Partly

Is the rationale for creating the dataset(s) clearly described?

Yes

Are the datasets clearly presented in a useable and accessible format?

Yes

Are the protocols appropriate and is the work technically sound?

Yes

Reviewer Expertise:

Bioinformatics

I confirm that I have read this submission and believe that I have an appropriate level of expertise to confirm that it is of an acceptable scientific standard.
